# Prevalence and determinants of tobacco use among university students in Lebanon: Implications for smoke-free campus policies

**DOI:** 10.1371/journal.pone.0361138

**Published:** 2026-10-22

**Authors:** Mohamad Ali Hijazi, Deema Rahme, Dana Jammoul, Lynn Al Jawiche, Mohamad Alameddine

**Affiliations:** 1 Head of Pharmaceutical Sciences Department, Faculty of Pharmacy, Beirut Arab University, Beirut, Lebanon; 2 Faculty of Pharmacy, Beirut Arab University, Beirut, Lebanon; 3 INSPECT-LB (Institut National de Santé Publique, d’Épidémiologie Clinique Et de Toxicologie-Liban), Beirut, Lebanon; 4 Faculty of Pharmacy, Beirut Arab University, Beirut, Lebanon; 5 Faculty of Health Sciences, Sharjah University, Sharjah, United Arab Emirates; Dana-Farber Cancer Institute, Harvard Medical School and THE Broad Institute of MIT and Harvard, LEBANON

## Abstract

**Background:**

The tobacco epidemic is a global public health threat, with addiction often starting in the early adulthood stage of life. The Middle East Region is slow in adopting smoke-free campus policies, and few studies have examined smoking attitudes and behaviors among university students and their perspective on such policies.

**Objective:**

The objective of this study was to determine the perceptions, knowledge, attitudes, and behaviors toward smoking (used to denote using any tobacco products) and smoke-free campus policies among university students aged between 17 and 30 years old at a large multicampus academic institution in Lebanon.

**Methods:**

The study utilized a comprehensive cross-sectional study design. The questionnaire used in this study included validated tools for assessing smoking behaviors, such as the Global Adult Tobacco Survey (GATS); the Youth Tobacco Survey (YTS); and the Smoking Knowledge, Attitudes, and Practices (KAP) survey, as well as sections for investigating attitudes toward smoking and the smoke-free campus policy.

**Results:**

This study reports an alarming prevalence of smoking on campuses (32.2%), with more than half of the surveyed smokers reporting attempts to quit smoking in the past 12 months (53.4%). The factors significantly associated with smoking included having family (OR 7.08, 95% C.I (2.83–17.69) or friends (OR 12.20, 95% C.I (5.48–19.76)) who smoke, as well as experiencing social, emotional, or mental distress (OR 4.09, 95% C.I (1.74–9.63)). Further analysis revealed that agreement with a smoke-free university policy was a significant factor associated with reduced odds of smoking (OR 0.58 95% C.I (0.45–0.78)), whereas the perceived unfairness of smoking bans on campuses was associated with increased odds of smoking (OR 1.87, 95% C.I (1.34–2.61)).

**Conclusions:**

The results highlight a promising window of opportunity to implement evidence-based interventions to support students in quitting smoking. Interventions include educational programs to raise awareness of the dangers of tobacco use and programs to break the association between exposure to stress and smoking. Such programs could include a combination of awareness programs and organizing stress-reducing services, workshops, and consultations. Because the design is cross-sectional, these findings describe associations rather than causal effects; they nonetheless support the further evaluation of smoke-free campus policies as one component of a broader strategy to lower students’ smoking rates, mitigate peer influence and foster healthier campus environments.

## Background

With more than 1.18 billion people referred to as regular smokers, the tobacco epidemic is one of the greatest public health threats worldwide [1]. According to the World Health Organization (WHO), smoking (used from this point forward to denote using any tobacco products) kills approximately eight million people annually, of which 1.2 million are due to exposure to secondhand smoke [2]. Active smoking is associated with numerous health concerns, most notably, twelve distinct types of cancer (mainly lung cancer), chronic obstructive pulmonary disease (COPD), emphysema, cardiovascular hazards, and cerebral and peripheral vascular diseases [3–5]. In addition to physical health impacts, smoking negatively affects the social wellbeing of smokers by reducing their social interactions and relationships, as well as altering their psychological and cognitive processes [6,7]. It is also associated with numerous mental health consequences, such as mood, anxiety, depression, and trauma-related disorders [8].

The detrimental effects of smoking are not limited to health consequences but also cause a heavy economic burden worldwide [9]. This economic burden also affects middle-income and low-income countries, resulting in substantial losses due to tobacco use [9].

In younger individuals, especially adolescents, smoking is driven by psychosocial motives. Recent studies reported that among young adults smoking starts for multiple reasons, including enjoyment and satisfaction, feeling mature and independent, relieving stress, feeling more secure, and for arousing curiosity [10]. Furthermore, the presence of a smoker in the family and the influence of peer pressure and social circles are significant motivators for young individuals to initiate the smoking habit [11].

Many reports have shown that smoke-free policies effectively reduce secondhand smoke (SHS) exposure and consumption among nonsmokers, encourage quitting attempts, prevent light smokers from becoming heavy smokers, minimize smoking relapse among smokers, and reduce exposure to thirdhand smoke [12]. A study from Georgia reported that a comprehensive smoking ban was followed by an estimated 10–20% reduction in hospital admissions for acute coronary events in the general population in the first year after the ban [13].

Considering that 99% of adult cigarette smokers first start smoking before the age of twenty-six and that many occasional smokers transition to daily smokers during young adulthood, universities and colleges could play crucial roles in protecting students from active smoking and secondhand smoke [14]. Tobacco-free and smoke-free policies at universities have been effective in decreasing exposure to secondhand smoke, reducing cigarette consumption, increasing active smokers’ intention to quit, and increasing the likelihood of cessation [15,16]. Unfortunately, the number of universities in the Middle East that are tobacco free is lower than elsewhere globally, and no published studies have yet evaluated their effectiveness in the region [17].

Lebanon is a small middle-income country (area 10452 km^2^) in the Eastern Mediterranean Region with an estimated population of approximately six million citizens and an additional two million refugees [18]. The smoking rate in Lebanon is alarming, where it exceeded 38% among adults in 2020, which is the highest reported rate in the Middle East Region [19]. On average, a Lebanese smoker consumed 1,955 cigarettes in 2022 [20]. Smoking from Lebanon revealed that people from lower socioeconomic backgrounds had higher smoking rates, and the smoking prevalence increased among adolescents with poor parental relationships and those whose parents were smokers [21].

Education was an important predictor of cigarette smoking in Lebanon. Those with preparatory, primary or less than primary education is significantly more likely to smoke cigarettes than those with secondary education or higher [22]. For a decade, tobacco has been the top risk factor driving death and disability in Lebanon [23]. The country is third in the world in terms of cigarette consumption per capita, contributing to 3,500 annual deaths resulting from tobacco-related diseases [21]. The expenditure on tobacco in Lebanon is equal to that spent on recreation and furniture and is only 1% less than that spent on education [24]. It has been reported that ex-smokers’ and smokers’ absenteeism in Lebanon leads to a loss of production of $102.2 million per year.

A ban on smoking in public places in Lebanon was declared in 1993, but it was inadequately enforced, leading to high smoking rates. In 1998, the Ministry of Public Health, in collaboration with the WHO, established the National Tobacco Control Program, and Lebanon ratified the WHO Framework Convention on Tobacco Control (FCTC) in December 2005. Finally, in 2011, the Lebanese Parliament passed a new tobacco-control act (Law 174), prohibiting smoking in all open public places, public transport, workplaces, and outdoor areas of education, health, and sports facilities. However, the act must be properly enforced [19]. Smoking further places a serious financial burden on families, especially with the multiple ongoing economic crises that Lebanon has encountered since October 2019 [25].

Among the forty-eight higher education institutions that exist in Lebanon [26], only a few universities have applied a successful smoke-free policy for students and staff [17]. Despite the release of tobacco control acts that prohibit smoking in closed areas, students and staff continue to smoke in various indoor and outdoor areas across campuses across the country.

The objective of this study was to determine the perceptions, knowledge, attitudes, and behaviors toward smoking among the students of a large multicampus university in Lebanon with 16000 students. The secondary objective was to determine the enablers of and barriers to establishing smoke-free campuses at this university.

## Methods

### Study design

This study utilized a cross-sectional design to evaluate tobacco use behaviors and attitudes among students from different faculties and different campuses at Beirut Arab University (BAU).

### Study setting

The investigation was conducted across the Beirut, Debbieh, and Tripoli campuses of BAU to ensure a representative geographic distribution of the student population. Data collection was performed electronically using the SurveyMonkey platform between 01/09/2023 and 31/12/2023. The survey instrument was available in both English and Arabic, with an average completion time of approximately 10 minutes. To ensure linguistic accuracy and conceptual equivalence, the questionnaire underwent a rigorous translation and back-translation process. Prior to full implementation, a pilot test was conducted with ten students to validate the questionnaire’s clarity and flow; no significant modifications were required following this phase.

### Study size

Sample size was determined based on the total university population according to the university database (approximately 16,000 students). Calculations indicated that a minimum sample of 317 students was required to detect a 30% smoking prevalence with a 95% Confidence Interval (CI) and a 5% margin of error.

### Participants and sampling

Eligible participants included students actively registered at any BAU faculty who were conversant in either English or Arabic. Recruitment was managed through official university communication channels to ensure broad and equitable access. Specifically, the survey link was disseminated via institutional email lists and integrated into university-wide digital platforms, including Moodle and iConnect. To ensure the sample accurately reflected the university’s geographic and academic diversity, a stratified random sampling method was employed, with the sample size allocated proportional to the size of each campus. A total of 480 students were invited to participate via direct email invitation. To mitigate non-response bias and reach the target response threshold, a second and third wave of invitation links were issued to the sampled population. In accordance with the approved Institutional Review Board (IRB) protocol, all respondents were required to provide electronic informed consent; individuals who were unwilling or unable to provide consent were excluded from the study.

In addition, to ensure data integrity and the completeness of the dataset, the digital questionnaire was configured to require responses for all items before the participant could progress to the next section. This setting was chosen to eliminate the risk of item-level missing data. Only individuals who successfully reached the final page of the survey and submitted their responses were included in the final analysis.

### Data sources and measurement

#### Questionnaire development and validation.

The questionnaire was developed by utilizing validated tools for assessing smoking behaviors such as the Global Adult Tobacco Survey (GATS), which is a standardized survey developed by the World Health Organization (WHO) [27]; the Youth Tobacco Survey (YTS), which was developed by the Centers for Disease Control and Prevention (CDC) [28]; and the Smoking Knowledge, Attitudes, and Practices (KAP) survey [29]. The validated tools were reviewed thoroughly, and the modified version of the questionnaire was subsequently developed by an expert panel of a pharmacist, an epidemiologist, and a public health professional. The questionnaire was a multi-component instrument designed to evaluate the behavioral and attitudinal landscape of tobacco use within the BAU community. The tool was organized into four sections to ensure a logical flow and minimize respondent fatigue, with the full instrument now provided in the Supplementary Appendix (S1 File).

#### Measurement of variables.

**The first section** captured socio-demographic and institutional variables, including participant’s age group, gender, and geographic campus location (Beirut, Debbieh, or Tripoli). Faculty affiliations collapsed into five major clusters: Medical, Sciences, Humanities, Engineering, and Others to facilitate comparative analysis.

**The second section** performed a comprehensive behavioral evaluation and usage pattern analysis by utilizing a filtering question to identify respondents as current smokers or non-smokers. For current smokers, the survey identified specific nicotine delivery systems, including combustible cigarettes, e-cigarettes, vapes, waterpipes/shisha, cigars, and pipes, to provide a granular descriptive analysis of usage. Usage metrics were further quantified by capturing the frequency of use, and the chronological onset of the habit to assess the depth of nicotine dependence. Additionally, the etiology of smoking was explored by asking participants to identify primary drivers for initiation, such as social influences (family or friends), environmental factors (media and advertisements), or psychological factors like emotional or mental distress. To facilitate regression modeling, these specific products were aggregated into a binary “Tobacco/Nicotine User” variable. This operationalization aligns with the study’s objective to inform a comprehensive “smoke-free” policy that regulates all forms of inhaled nicotine regardless of the device type.

**The third section** quantified health literacy by assessing the participants’ knowledge of tobacco-related morbidity, specifically asking if they believed smoking causes serious illnesses like stroke, cardiovascular diseases, lung cancer, and diabetes. This was operationalized by assigning one point for each correct identification, resulting in a cumulative knowledge score.

**Finally, the fourth section** utilized Likert-type scales to measure attitudes toward institutional policy, evaluating the perceived impact of a smoke-free mandate on student quality of life and learning, as well as the level of support for specific restrictions on vaping and the prohibition of tobacco sales on campus.

#### Variables.

The primary outcome variable was smoking status, operationalized as a binary variable (tobacco/nicotine user versus non-user). Predictors and potential confounders included:

- Socio-demographic Factors: Age, gender, campus location, and faculty affiliation (clustered into Medical, Sciences, Humanities, Engineering, and Others).
- Behavioral & Environmental Factors: Frequency of use, age of onset, primary drivers for initiation (e.g., social or psychological distress), and exposure to secondhand smoke.
- Knowledge & Attitudes scores: A cumulative knowledge score based on tobacco-related morbidity (e.g., stroke, lung cancer) and Likert-scale attitudes toward smoke-free policies.

#### Bias.

Different types of bias were addressed through several measures. Stratified random sampling was conducted via official university channels, and non-response bias was minimized by implementing three iterative waves of recruitment until the target sample size was achieved. To reduce information bias and ensure instrument validity, the questionnaire was reviewed by an expert panel, and it underwent a formal English-Arabic translation with back-translation. Pilot study confirmed the clarity of the tool and its completion time. Data integrity was safeguarded by employing a “forced response” design on the SurveyMonkey platform, which required participants to answer all items before progressing thereby ensuring the integrity of the dataset.

### Statistical methods

The data were analyzed via IBM SPSS Statistics version 27 [30]. Descriptive statistics were calculated for all the variables. Categorical variables are presented as frequencies and percentages, whereas continuous variables are presented as means and standard deviations. Inferential statistical tests were selected based on the research questions and the type and distribution of the variables. Continuous variables were tested for normality via visual inspection of histograms, skewness and kurtosis, and the Shapiro‒Wilk test. Bivariate analysis utilized Pearson chi-square tests to examine the associations between smoking status and categorically independent variables, including Faculty, Age, Gender, Knowledge of university smoking policy, Exposure to secondhand smoke on campus, University-provided education on tobacco hazards, Presence of smokers among family members or friends, and Social, emotional, and mental distress.

Student’s t tests were used to compare knowledge assessment and policy agreement scores between smokers and nonsmokers. Multivariable logistic regression with backward stepwise elimination was conducted to identify significant predictors of smoking status.

The variables with a p-value of less than 0.2, including significant ones from the bivariate analysis, were included in the logistic regression model to identify the significant factors associated with being smokers after adjusting for the other variables. Adjusted odds ratios with 95% confidence intervals were calculated to quantify the associations. Model fit was assessed using the omnibus test of model coefficients, the Hosmer–Lemeshow goodness-of-fit test and the Nagelkerke pseudo-R²; the latter is reported as an index of relative model fit and not as the proportion of variance explained. A p-value of less than 0.05 was considered statistically significant. For the purposes of the regression analysis, various forms of tobacco and nicotine delivery systems including combustible cigarettes, waterpipes (shisha), and electronic nicotine delivery systems (e-cigarettes/vapes) were aggregated into a single ‘smoking/nicotine use’ variable. This approach was chosen because the study’s primary objective was to assess barriers and enablers for a comprehensive smoke-free campus policy. Since such policies typically prohibit all forms of inhaled tobacco and nicotine products on university grounds regardless of the device type, grouping these behaviors allowed for a more policy-relevant analysis of student attitudes.

### Ethical considerations

Ethical approval was obtained from the Institutional Review Board (IRB) at the BAU (protocol number 2023-H-0090-P-R-0541). The participants were asked to electronically sign the consent form before completing the questionnaire. Participation was entirely voluntary, and there was no risk or harm resulting from completing the questionnaire. All ethical concerns related to participants’ anonymity and confidentiality were assured in this study.

## Results

### Demographics and the use of any tobacco products status evaluation

A total of 407 surveys were completed out of 480 distributed, corresponding to a response rate of 85%. This final sample size exceeded the calculated minimum required sample size of 317 participants.

As represented in Table 1, the study included 407 students. Most participants were medical students (56.8%), and most of them were 20–30 years old (63.6%), with a slightly greater percentage of female participants observed (54.8% females, 45.2% males).

**Table 1:** Sociodemographic Characteristics of the Study Participants (N = 407).

Category		n	%
Faculty	Medical	231	56.8
	Humanities	60	14.7
	Engineering	51	12.5
	Sciences	40	9.8
	Others	25	6.1
Campus	Beirut	323	79.4
	Debbieh	62	15.2
	Tripoli	22	5.4
Age (years)	Below 20	148	36.4
	20-30	259	63.6
Gender	Males	184	45.2
	Females	223	54.8

Percentages are calculated based on the total sample size (N = 407).

As depicted in Table 2, close to one-third of the students participating were users of tobacco products (32.2%), of whom more than half started to use these products before 18 years of age (55.0%). Among tobacco users, the most common frequency was 11–20 times daily (33.6%), followed by more than twenty times daily (18.3%), indicating heavy tobacco use. Most of the tobacco users found it easy (98.5%) and affordable (90.1%) to obtain their products. Use of tobacco on campus was reported by 71.8% of smokers, and 65.1% of all students reported being exposed to secondhand smoke on campus multiple times daily.

**Table 2:** Smoking Status and Smoking Characteristics (N = 407).

Variable	Category	n	%
Smoking status	Non-smoker	276	67.8
	Smoker	131	32.2
Frequency of tobacco use (Smokers only, N = 131)	<2 times/day	25	19.1
	2–5 times/day	17	13.0
	6–10 times/day	21	16.0
	11–20 times/day	44	33.6
	>20 times/day	24	18.3
Age at smoking initiation (Smokers only)	<18 years	72	55.0
	≥18 years	59	45.0
Use tobacco on campus (Smokers only)	Yes	94	71.8
	No	37	28.2
Ease of purchasing tobacco (Smokers only)	Easy	129	98.5
	Difficult	2	1.5
Affordability of tobacco (Smokers only)	Affordable	118	90.1
	Not affordable	13	9.9
Exposure to secondhand smoke on campus (All students)	Never	42	10.3
	Monthly	10	2.5
	Weekly	36	8.8
	Once daily	54	13.3
	Multiple times daily	265	65.1

Percentages calculated within the appropriate subgroup.

The students reported that the most common type of smoking was cigarettes (37.3%), as illustrated in S1 Fig. Furthermore, the most common places to smoke on campus were outside buildings (32.3%), followed by cafeterias (22.9%), as demonstrated in S2 Fig.

### Smokers’ intentions to quit

Among the 131 smokers surveyed, 53.4% (n = 70) had attempted to quit smoking in the past 12 months. This suggests that a substantial proportion of smokers are actively trying to quit, with the most common reasons being improved health (53.4%), fear of addiction (20.6%), and financial savings (10.7%).

Smokers identified several barriers to quitting, including perceived stress relief (26.0%), social and environmental factors (21.4%), addiction and dependence (32.1%), and lack of motivation (20.6%). These findings suggest that successful cessation requires addressing both individual psychosocial factors and the broader social context in which smoking occurs.

Interestingly, 30.5% (n = 40) of smokers reported that warning labels on cigarette packages had led them to consider quitting. This highlights the potential of simple public health interventions to influence smokers’ attitudes toward cessation.

The smokers’ self-reported likelihood of quitting varied widely, with 42% reporting a willingness to quit. This suggests that while some smokers are highly motivated to quit, others may require more intensive intervention to overcome barriers and make a sustained quit attempt. If smokers were to try quitting within the next 30 days, 48.1% believed it would be successful. These findings indicate that while a portion of smokers are confident in their ability to quit, many may need additional support and resources to achieve long-term cessation. The results are illustrated in Table 3.

**Table 3:** Smoking Cessation Attempts, Motivations, Barriers, and Perceptions (Smokers only, N = 131).

Variable	Category	n	%
Tried to quit in past 12 months	Yes	70	53.4
	No	61	46.6
Reason for quitting*	Improved health	70	53.4
	Fear of addiction/dependence	27	20.6
	Financial savings	14	10.7
	Improved taste/smell	14	10.7
Barriers to quitting*	Addiction/dependence	42	32.1
	Perceived stress relief	34	26.0
	Social/environmental factors	28	21.4
	Lack of motivation	27	20.6
Warning labels encourage quitting	Yes	40	30.5
	No	91	69.5
Likelihood of quitting currently	Very likely	24	18.3
	Likely	31	23.7
	Undecided	38	29.0
	Unlikely	20	15.3
	Very unlikely	18	13.7
Perceived success if quitting in 30 days	Very likely	40	30.5
	Likely	23	17.6
	Undecided	39	29.8
	Unlikely	17	13.0
	Very unlikely	12	9.2

* Multiple responses allowed.

### Students’ Awareness and Preferences Regarding University Smoking Policies

Less than half of the participating students (48.4%, n = 197) reported being aware that the university had a policy prohibiting smoking inside campus buildings, whereas a slightly higher proportion (51.6%, n = 210) indicated no such awareness.

Regarding preferences for university smoking policies, nearly half of the students (48.6%, n = 198) favored a policy that allows smoking outdoors only in designated areas. Approximately one quarter of respondents (23.3%, n = 95) supported a comprehensive smoke-free policy that prohibits smoking in all campus locations. In contrast, 21.6% (n = 88) preferred allowing smoking outdoors across all campus areas, while only a small minority (6.4%, n = 26) supported a policy permitting smoking inside campus buildings. This underscores a general preference for no indoor smoking, which is consistent with the harms of secondhand smoke and the importance of providing a healthy environment to all members of the university community.

### Students’ Knowledge of the Dangers of Tobacco Use

As shown in Table 4, most students (68.1%, n = 277) reported having received instruction at their institution regarding the dangers of tobacco use. High levels of awareness were observed concerning the association between smoking and major illnesses, including cardiovascular diseases (81.3%) and lung cancer (93.4%). Furthermore, most students recognize that smokeless tobacco use can cause serious illness. In contrast, a substantial proportion of students (77.6%) indicated that smoking does not cause diabetes. The mean knowledge score among participants was 4.79 ± 0.93, with scores ranging from 3 to 6.

**Table 4:** Students’ Knowledge of Tobacco-Related Health Risks (N = 407).

Knowledge item	Yes n (%)	No n (%)
1. Smoking causes serious illness	382 (93.9) *	25 (6.1)
2. Smoking causes cardiovascular disease	331 (81.3) *	76 (18.7)
3. Smoking causes lung cancer	380 (93.4) *	27 (6.6)
4. Smoking causes diabetes	91 (22.4) *	316 (77.6)
5. Smoking does not cause any disease	7 (1.7)	400 (98.3) *
6. Smokeless tobacco causes serious illness	297 (73.0) *	110 (27.0)
Taught about tobacco dangers at university	277 (68.1)	130 (31.9)

* Denotes the correct answer.

### Bivariate analysis

Among the students who smoked, a greater proportion were enrolled in medical faculties (55.7%) than in nonmedical faculties (44.3%). These figures describe the distribution of smokers across faculties rather than the prevalence of smoking within each faculty, and faculty affiliation was not significantly associated with smoking status (p = 0.831). Age was significantly associated with smoking status (p < 0.001): 22.9% of smokers were under 20 years of age and 77.1% were aged 20–30 years. Expressed as within-group prevalence, 20.3% (30/148) of students under 20 years were smokers, compared with 39.0% (101/259) of those aged 20–30 years. Gender was also significantly associated with smoking status (p < 0.001): 63.4% of smokers were male and 36.6% were female, corresponding to a within-group prevalence of 45.1% (83/184) among male students and 21.5% (48/223) among female students. Knowledge of the university anti-smoking policy was significantly associated with smoking status (p = 0.004): 58.8% of smokers, compared with 43.5% of nonsmokers, reported being aware of the policy. Exposure to secondhand smoke on campus was also significantly associated with smoking status (p < 0.001): 80.2% of smokers, compared with 58.0% of nonsmokers, reported such exposure multiple times daily, whereas students who were never exposed accounted for only 2.3% of smokers. Furthermore, 68.1% of the students reported having been taught about the dangers of tobacco use at their institution, but this did not differ significantly by smoking status (p = 0.088).

Moreover, the study explored smoking-related factors among university students. The key findings included a strong influence of peer pressure, with students who reported having friends (60.3%) or family members who were smokers (35.1%) being significantly more likely to smoke (p < 0.001). Exposure to smoking advertisements did not significantly predict smoking status (p = 0.075). Interestingly, students who experienced social, emotional, or mental distress were significantly more likely to smoke (59.5%, p < 0.001).

The study also investigated the students’ perceptions of smoking. Students who smoked were considerably less likely than nonsmokers to describe the image of a smoker as desirable (6.9% vs. 40.2%; p < 0.001). The study examined students’ perceptions of the taste and smell of smoking. A vast majority of nonsmokers (94.2%) and smokers (77.1%) reported that they did not like the taste and smell of smoke. However, a small percentage of students (5.8% of nonsmokers and 22.9% of smokers) reported that smoking was associated with a good taste or smell (p < 0.001). In contrast, the perceived affordability of smoking products was not significantly associated with smoking status (p = 0.619).

Furthermore, the study compared preferences for university smoking policies between nonsmokers and smokers. Among nonsmokers, a majority (53.3%, p < 0.001) favored a policy that allowed smoking outdoors at specific locations only. This approach was significantly more popular than were policies permitting smoking outdoors anywhere on campus (13%) or those allowing smoking indoors (2.2%). However, the most popular policy chosen by smokers was the one that allows outdoor smoking anywhere on campus (39.7%), followed by policies that allow smoking at specific locations (38.9%) and indoors (15.3%).

The effects of implementing a smoke-free policy on various aspects of university students’ lives revealed significant improvements in students’ quality of life, learning, and enrollment (p < 0.001) following the implementation of the policy. Compared with smokers, nonsmokers agreed more strongly with the importance of smoke-free environments and the dangers of smoking to both smokers and nonsmokers (p < 0.001). However, smokers significantly agreed on the difficulty of enforcing a smoke-free policy (p < 0.001) and reported that a smoke-free policy may not affect their smoking behavior (p < 0.001).

Finally, when the knowledge scores of nonsmokers and smokers were compared, nonsmokers had a higher average knowledge score (M = 4.68, p = 0.274) than smokers did (M = 4.50), but the result was not as significant as the previous results. The detailed results are outlined in Table 5.

**Table 5:** Bivariate Analysis of Factors Associated with Smoking Among University Students.

	Variable		Nonsmokers (N=276) n (%) Mean± SD	Smokers (N=131) n (%) Mean± SD	*p-value*
	Faculty	Medical	158 (57.2%)	73 (55.7%)	0.831^a^
		Non-Medical	118 (42.8%)	58(44.3%)	
	Age	Below 20	118 (42.8%)	30 (22.9%)	< 0.001^a^*
		20-30	158 (57.2%)	101 (77.1%)	
	Gender	Males	101(36.6%)	83 (63.4%)	< 0.001^a^*
		Females	175(63.4%)	48 (36.6%)	
	Knowing the university policy against smoking	Yes	120 (43.5%)	77 (58.8%)	0.004^a^*
		No	156(56.5%)	54 (41.2%)	
	Exposure to smoke from smokers on campus	Never	37 (13.4%)	3 (2.3%)	< 0.001^a^*
		On monthly basis	7 (2.5%)	3 (2.3%)	
		On weekly basis	36 (13%)	2 (1.5%)	
		Once daily	36 (13%)	18 (13.7%)	
		Multiple times daily	160 (58%)	105 (80.2%)	
	Were you taught in your institution about the dangers of tobacco use?	Yes	180 (65.2%)	97 (74%)	0.088^a^
		No	96 (34.8)	34 (26%)	
Smoking related factors	Family members are smokers	No	234(84.8%)	85 (64.9%)	< 0.001^a^*
		Yes	42(15.2%)	46 (35.1%)	
	Friends are smokers	No	221(80.1%)	52(39.7%)	< 0.001^a^*
		Yes	55(19.9%)	79 (60.3%)	
	Media and smoking advertisements	No	270(97.8%)	123(93.9%)	0.075^a^
		Yes	6 (2.2%)	8 (6.1%)	
	Social, emotional, and mental distress	No	230 (83.3%)	53 (40.5%)	< 0.001^a^*
		Yes	46 (16.7%)	78 (59.5%)	
	Desirable image of smokers	No	165 (59.8%)	122 (93.1%)	< 0.001^a^*
		Yes	111 (40.2%)	9 (6.9%)	
	Affordability of smoking products	No	25 (9%)	13 (9.9)	0.619^a^
		Yes	251 (91%)	118(90.1%)	
	Good taste/smell of smoking	No	260 (94.2%)	101 (77.1%)	< 0.001^a^*
		Yes	16(5.8%)	30 (22.9%)	
Policy	Which type of university policy would be the best for the college community?	A policy that always prohibits smoking	87 (31.5%)	8 (6.1%)	< 0.001^a^*
		A policy that allows smoking outdoors at specific locations only	147 (53.3%)	51 (38.9%)	
		A policy that allows smoking outdoors on campus in all locations	36 (13%)	52 (39.7%)	
		A policy that allows smoking inside campus buildings	6 (2.2%)	20 (15.3%)	
	The effect of implementing a smoke-free policy on the students’ Quality of Life?		3.74 ± 1.228	2.38 ± 1.262	< 0.001^b^*
	The effect of implementing a smoke-free policy on the students’ Learning?		3.58± 1.098	2.52 ±1.166	< 0.001^b^*
	The effect of implementing a smoke-free policy on the students’ Enrollment?		3.3 ±1.126	2.59 ±1.014	< 0.001^b^*
	How likely do you support a Smoke-Free campus?		4 ±1.204	2.83 ±1.525	< 0.001^b^*
	How likely do you support Restrictions for e-cigarettes/vaping on campus?		3.93 ±1.229	2.77±1.476	< 0.001^b^*
	How likely do you support the Ban on tobacco sales on campus?		4 ±1.308	2.97±1.483	< 0.001^b^*
	How much do you agree that smoking ban is unfair?		2.84±1.123	4.02±1.023	< 0.001^b^*
	How much do you agree that smoking is dangerous to health?		4.61 ±0.809	4.15 ±0.993	< 0.001^b^*
	How much do you agree that a smoke-free policy is difficult to enforce?		3.71 ±1.040	4.06 ±0.990	< 0.001^b^*
	How much do you agree that a smoke-free campus helps smokers quit?		3.26±1.195	2.23 ±1.161	< 0.001^b^*
	How much do you agree that smoking is dangerous to Nonsmokers’ health?		4.4 ±0.768	3.67 ±1.041	< 0.001^b^*
	How much do you agree that public places should be smoke-free?		3.76 ±1.103	2.66 ±1.346	< 0.001^b^*
	How much do you agree that passive smoking shortens life?		4.02 ±0.878	3.31 ±1.183	< 0.001^b^*
	How much do you agree that designated smoking areas should be assigned?		3.83 ±1.052	3.49 ±1.230	0.006^b^*
Knowledge	Knowledge score		4.68 ±0.939	4.50 ±1.303	0.274^b^

* Denotes a significant p value < 0.05 (a-Pearson chi-square test, b-Student t test).

### Multivariable logistic regression

The study revealed statistically significant results regarding the factors associated with smoking behaviors among university students, where the dependent variable was smoking status (smoker or non-smoker), with non-smokers serving as the reference group.

The regression model included variables with a p-value of less than 0.2 from the bivariate analysis, which were: age, gender, awareness of the university policy against smoking, exposure to secondhand smoke from smokers on campus, having smokers among family and friends, and social distress. Additionally, several proposed university policies were included in the model.

Notably, students who reported having family or friends who smoke, as well as those experiencing social, emotional, or mental distress, demonstrated significantly increased odds of being current smokers, with adjusted odds ratios of 7.077, 12.203, and 4.091, respectively. In contrast, students who described the image of a smoker as desirable had markedly lower adjusted odds of being smokers (AOR 0.03, 95% CI 0.01–0.10); this unexpected association is considered in the Discussion.

Further analysis revealed that agreement with a smoke-free university policy was a significant factor associated with reduced odds of smoking (OR 0.589), whereas the perceived unfairness of smoking bans on campuses was associated with increased odds of smoking (OR 1.869). Importantly, the statistical significance of each factor was underscored by a p value of less than 0.05, further emphasizing the robustness of these associations, as demonstrated in Table 6.

**Table 6:** Multivariable Binary Logistic Regression Analysis of Factors Associated with Smoking Among University Students.

Predictor Variable	Reference category	β	SE	AOR	95% C. I	*p-*value
Family members are smokers	No	1.98	0.49	7.08	2.83–17.70	<0.001*
Friends are smokers	No	2.50	0.41	12.20	5.48–19.76	<0.001*
Social, emotional, or mental distress	No	1.79	0.49	4.09	1.74–9.63	0.001*
Desirable image of a smoker	No	−4.37	0.67	0.03	0.01–0.10	<0.001*
Adoption of a smoke-free university policy ¥	Per 1-unit increase	−0.47	0.15	0.59	0.45–0.78	0.002*
Perception that smoking-ban policy is unfair ¥	Per 1-unit increase	0.68	0.19	1.87	1.34–2.61	<0.001*

**AOR** adjusted odds ratio, **B** coefficient for the constant in the null model, **CI** confidence interval, **SE** standard error.

Multivariable logistic regression analysis was conducted via backward stepwise elimination. The omnibus test of model coefficients was significant (p < 0.001) and the Hosmer–Lemeshow goodness-of-fit test was non-significant (p = 0.341). The Nagelkerke R² was 0.69, indicating good model fit.

¥ Continuous scale variables.

* Statistically significant (*p-value* < 0.05).

## Discussion

This study presents a rare attempt to examine the perceptions, knowledge, attitudes, and behaviors toward smoking among university students in Lebanon and to determine the enablers of and barriers to establishing smoke-free campuses among university students.

The study reported an alarmingly high prevalence of smoking inside campuses, along with high percentages of secondhand smokers. Moreover, most students started smoking after they joined the university, with the results revealing that obtaining smoking products was very easy and affordable for students. On the other hand, the findings of this study provide a window of opportunity for proper intervention, communication, and support for students not to become smokers (if they are nonsmokers), to quit smoking (if they are smokers), and to manage their social, emotional, and mental distress to have better health, improve their university life, and quality of life. The strong influence of peer pressure was evident in this study. Furthermore, the results revealed a positive perception of the smoke-free policy among university students, but its implementation may present challenges that need to be addressed.

The fact that one of every three surveyed university students indicated that they are active smokers is quite concerning and highlights a clear prevalence of smoking among students. An examination of the reported smoking prevalence among university students in other countries revealed that the rate in our study was higher than that reported among college students in the KSA (17%) [31] and college students in Iran (11%) [32]. This rate is, however, lower than that reported among college students in Egypt (46.7%) and Kuwait (46%) [33]. The findings make it clear that Lebanese university students have a high smoking rate and that the burden of disease from early smoking habits is expected to be significant and overburden the healthcare system in the future. The results also underscore a window of opportunity for organizations’ evidence-based programs and interventions aimed at helping students quit this harmful habit during their university years. Examples of such programs include many smoking cessation programs that provide students with access to consultations with specialized physicians along with the prescription of nicotine and no nicotine medications, behavioral counseling sessions with a certified smoking cessation counsellor with a 1-year follow-up to maintain progress, and dietetic counseling for adequate meal planning and weight management. In addition, group-based interventions offer patients the opportunity for social learning; for example, exchanging skills and knowledge about behavioral techniques for smoking cessation would create emotional experiences and provide mutual support [34]. Banning the sale of tobacco and tobacco products on and off campus could also be beneficial, as it can aid in reducing the number of illnesses and deaths caused by smoking [35], especially that 65% of nonsmoking students are identified as secondhand smokers.

Furthermore, the fact that most smokers were in the 20–30 years of age group may indicate that the smoking habits of university students started after they joined university, which further supports the importance of organizing antismoking awareness and intervention programs. The design of educational campaign interventions among young people clearly led to preventive smoking behaviors, causing lifestyle changes, preventing health problems, reducing treatment costs, and increasing quality of life and life expectancy [36].

The study further revealed an opportunity to enhance the education of university students about the perils of smoking. Specifically, there is a clear lack of knowledge of the negative effects of smokeless tobacco, as well as the association between smoking and some chronic diseases (e.g., type 2 diabetes mellitus). Our findings are in accordance with those of previous studies that reported that students consistently rated smokeless tobacco as less likely than cigarettes to lead to long-term chronic disease, with the notable exception of oral cancer [37], as well as inadequate knowledge about the harmful effects of smoking [38]The fact that 53% of smokers reported that they had tried to quit in the past 12 months is another opportunity to build on their willingness to quit and underscore the need for effective intervention to support their attempts.

Interestingly, the study revealed that the odds of smoking increased by 12-fold if the student had friends who were smokers, highlighting the central effect of peer pressure on smoking habits. This fact was also highlighted in previous studies where higher odds of cigarette smoking among students who experienced peer pressure were reported than among those who did not [39], as was the influence of friends being one of the major reasons people started smoking at a young age [10].

The study revealed that exposure to smoking environments on campus was significantly associated with students’ smoking status. Therefore, implementing smoke-free policies on university campuses has emerged as a crucial strategy to mitigate this influence and reduce student smoking rates. By either completely prohibiting smoking or confining it to designated areas, campuses can create environments that discourage smoking initiation and support cessation efforts among current smokers. Such measures are expected to alleviate the pressure on nonsmoking students to conform to smoking norms and minimize their exposure to harmful secondhand smoke. This approach not only promotes a healthier campus atmosphere but also aligns with public health recommendations aimed at reducing tobacco-related harm among young adults [40,41].

The results further indicate a differential in support of implementing a smoke-free policy on campus, with a natural inclination of smokers to have lower support or to resist the implementation of such policies. To highlight this point, our results revealed that students who agreed with the implementation of a smoke-free university policy are close to half the odds of being smokers. In contrast, students who thought that a smoking ban policy was unfair were close to double the odds of being smokers.

A key finding worth noting is that smokers were far less likely than non-smokers to view the image of a smoker as desirable (6.9% vs. 40.2%). There are two likely reasons for this gap. First, it could be a psychological defense mechanism. Most smokers in this study knew tobacco is harmful, and over half had recently tried to quit. Calling the smoker image undesirable helps them deal with the guilt of continuing a habit they dislike, especially in a health-focused university setting that discourages smoking. Second, the two groups likely interpret the question differently: non-smokers base their answer on abstract media portrayals, while smokers base theirs on real, daily experience. Finally, because this data was collected at a single point in time, we can only say this attitude is linked to smoking, not that it causes it.

Finally, students indicate that smoking helps them deal with their social, emotional, and mental distress. This is a serious misconception that lures many students to smoke during their university years. It is essential to debunk this misconception and break this association through a combination of offering awareness programs and organizing stress-reducing services, workshops, and consultations. Examples of similar programs include the Mindfulness-Based Stress Reduction (MBSR) [42] and Cognitive Behavioral Therapy (CBT) programs [43]. Both programs provide individuals with strategies to overcome challenges and increase their chances of quitting successfully [44]. MBSR integrates mindfulness meditation and yoga to increase awareness of thoughts and emotions, helping smokers manage stress and cravings more effectively and thus reducing the risk of relapses. On the other hand, CBT focuses on identifying triggers, altering negative thought patterns, and teaching coping strategies to resist cravings [45]. By addressing both the psychological and behavioral aspects of smoking addiction, these programs equip individuals with practical tools to achieve and maintain abstinence from smoking. Research consistently supports their efficacy in improving quit rates and promoting long-term smoking cessation [46].

This study has several potential shortcomings that are worth mentioning. First, the cross-sectional nature of the study supports the establishment of associations but does not permit inferences about causality or temporal ordering. This caveat applies with particular force to the attitudinal and perceptual variables retained in the multivariable model — agreement with a smoke-free campus policy, the perception that a smoking ban is unfair, and the perceived desirability of the image of a smoker — which are at least as plausibly consequences of smoking as they are antecedents of it. These estimates should therefore be read as characterizing the attitudinal profile that accompanies student smoking rather than identifying modifiable causes of it. Second, the study was carried out at a single academic institution, albeit across its three campuses in Beirut, Debbieh and Tripoli, which may affect the external validity of the findings. Third, despite assurances that the study protects the confidentiality of respondents, the authors are unable to ascertain the absence of social desirability bias. Fourth, medical and health students were overrepresented in the responses, which could skew the findings of this study toward greater knowledge about the dangers of smoking. Lastly, this study grouped different tobacco and nicotine products for regression analysis. While we provided a descriptive breakdown of product types, we recognize that the health risks and social perceptions of these products differ. However, given our focus on an all-inclusive campus smoking ban, this grouping was necessary to evaluate general compliance and support for the policy. Future research could investigate whether attitudes toward campus restrictions vary significantly between traditional cigarette smokers and other types.

## Conclusion

The study discussed a serious public health problem among young people (university students) in an attempt to explore the associated factors, barriers, and necessary interventions to protect university students from serious health problems related to smoking. The results highlight a promising window of opportunity to implement evidence-based interventions to support students in quitting smoking. Interventions include educational programs to raise awareness of the dangers of tobacco use and programs to break the association between exposure to stress and smoking. Such programs could include a combination of awareness programs and organizing stress-reducing services, workshops, and consultations. Because the study is cross-sectional, the relationships reported here are associations rather than causal effects. They nonetheless support the further evaluation and implementation of smoke-free campus policies as one component of a broader strategy to lower students’ smoking rates, mitigate peer influence and foster healthier campus environments.

## Supporting information

S1 File Study Questionnaire. (DOCX)

S1 Fig Types of tobacco and nicotine products used by the students who reported smoking (N = 131). Percentages are calculated within the smoker subgroup.
(DOCX)

S2 Fig Locations on campus at which the students who reported smoking (N = 131) stated that they smoke. Percentages are calculated within the smoker subgroup.
(TIF)
