# Supplementary material for: Prevalence and determinants of tobacco use among university students in Lebanon: Implications for smoke-free campus policies
Source: PLoS One. 2026 Oct 22;21(10):e0361138. doi: 10.1371/journal.pone.0361138 (PMC13690209; doi:10.1371/journal.pone.0361138)
Supplement: Supplementary file 2 [file pone.0361138.s002.docx]

**Knowledge, Attitudes, and Behaviors towards Smoking at BAU community: Enablers and Barriers to create Smoking Free Campuses**

Questionnaire

**Section 1: Socio-demographic characteristics:**

1. To which Campus do you belong:
   - Beirut Campus
   - Debbieh Campus
   - Tripoli Campus
2. To which faculty does your study or field belong to:

- Medical (medicine, pharmacy, dentistry, Health sciences)
- Sciences
- Humanities
- Engineering
- Others

3. Age:

- Below 20
- 20 - 30

4. Gender:

- Male
- Female

**Section 2: Smoking Status Evaluation:**

1. Are you a smoker including any type of smoking such as: cigarettes, e-cigarettes, cigars, Vape, Pipe and Shisha: [ each choice should drive the participant to his/her specific section]

- Smoker
- Non-smoker (skip to question A-page 5)

2. How often does someone smoke beside you on campus?

- Multiple times daily
- Once daily
- On weekly basis
- On monthly basis
- Never

**IF Smoker:**

1. What type of smoking do you use? (you can choose more than one)

- Cigarettes
- e-cigarettes
- cigars
- Vape
- Pipe
- Shisha
- Roll-your-own
- Others; ___________________________

2. How often do you smoke?

- Less than 2 times daily
- From 2-5 times daily
- From 6-10 times daily
- From 11-20 times daily
- More than 20 times daily

3. When did you start smoking?

- Before 18 years old
- 18 years old and above

4. Why did you start smoking? (You can choose more than one)

- Because my family are smokers
- Because of my friends are smokers
- Because of media and smoking Advertisements
- Because of social, emotional, mental distress
- Because of the image of a smoker
- Others ___________________

5. Do you smoke inside the campus:

- Yes
- No

6. During the past 12 months, have you tried to stop smoking?

- Yes
- No

7. a. If yes, why did you try to stop smoking? (select all that applies)

- Improved health
- Fear of addiction/dependence
- Financial savings
- Improved taste/smell
- Other ----------

7.b. If No, why did NOT you try to stop smoking? (select all that applies)

- Addiction/dependence
- Perceived stress relief
- Social/environmental factors (smoking friends or family members, hanging out in smoking places)
- Lack of motivation
- Other ------

8. In the last 30 days, have warning labels on cigarette packages led you to think about quitting?

- Yes
- No

9. Is it easy for you to get your smoking product?
   - Easy
   - Difficult
10. How affordable is it for you to get your smoking product:

- Affordable
- Not affordable

11. Currently, how likely are you to stop smoking?

Likert scale

- Very likely – likely – undecided – unlikely – very unlikely

12. If you try to quit smoking within the next 30 days, how likely you believe that you will be successful?

- Very likely – likely – undecided – unlikely – very unlikely

**IF Non-Smoker**

A- Why didn't you become a regular smoker? (Select all that applies)
   - I did not like the taste and/or the smell of cigarettes
   - I did not want to become addicted to nicotine
   - I was concerned about the effects of smoking on my health
   - I was discouraged to smoke because my friends are non-smokers
   - I did not like the image of being a smoker
   - I don’t have social or mental distress
   - It was hard for me to obtain cigarettes
   - I could not afford to buy cigarettes
   - I was not encouraged by media and smoking advertisements
   - Since I grew up in a non-smoking family

**Section 3: Knowledge/Beliefs: (Questions apply to smokers and non-smokers)**

1. Based on what you know or believe, does smoking tobacco cause serious illness?

- Yes
- No

2. Based on what you know or believe, does smoking cause any of the following? You can choose more than one option.

- Cardiovascular diseases
- Lung cancer
- Diabetes
- None

3. Based on what you know or believe, does using smokeless tobacco cause serious illness?

- Yes
- No

4. Where you taught in your institution about the danger of tobacco use?

- Yes
- No

**Section 4: Attitudes towards smoking free campus: [ for ALL]**

1. What effect, if any, do you think a policy making this campus completely smoke-free would have on

- Student quality of life
- Student learning
- Student enrollment

Likert-type scale (1 = extremely negative 2 = negative 3 = neutral 4 = positive 5 = extremely positive)

2. How likely do you support?

Likert scale (1=Extremely Unlikely, 2=Somewhat Unlikely, 3=Likely, 4=Somewhat Likely, 5=Extremely Likely)

(a) An aspiration for smoke-free campus
(b) Restrictions for e-cigarettes/vaping on campus
(c) A ban of tobacco sales on campus.
3. Which type of university-use policy do you believe would be best for the college community?

- A policy that allows smoking inside campus buildings
- A policy that allows smoking outdoors on campus in all locations
- A policy that allows smoking outdoors at specific locations only
- A policy that always prohibits smoking anywhere on campus

4. How much do you agree with the following statements?

5=Strongly agree – 4=Agree – 3=Neutral – 2=Disagree – 1=Strongly disagree

- A university should be a smoke-free environment.
- A smoking ban would be unfair to smokers.
- Smoking is dangerous to smokers’ health.
- A smoke-free policy is difficult to enforce.
- A smoke-free campus will help a smoker quit.
- Tobacco smoke is dangerous for nonsmokers’ health.
- Public places should be smoke free.
- Smoke from other people’s cigarettes could shorten my life.
- Designated areas for smoking should be assigned in the university.

5. Do you know if the university has any policy against smoking in buildings?

- Yes, the university has a policy
- No, the university does not have a policy
- Not sure if there is a policy

6. Where do you see people smoke at the university? [you can choose more than one option]

- In offices
- In stairwells or corridors
- In lounges or student areas
- In classrooms or lecture halls
- In restrooms
- In university restaurants or cafeterias
- Outside the buildings
